# Seroprevalence Study of Human Brucellosis by Conventional Tests and Indigenous Indirect Enzyme-Linked Immunosorbent Assay

**DOI:** 10.1100/2012/104239

**Published:** 2012-04-01

**Authors:** Annapurna S. Agasthya, Srikrishna Isloor, Prabhudas Krishnamsetty

**Affiliations:** ^1^Department of Biotechnology, Lovely professional University, Punjab 144402, India; ^2^Department of Veterinary Microbiology, Veterinary College, KVAFSU, Bangalore 560024, India; ^3^Project Directorate on Animal Disease Monitoring and Surveillance, Indian Council of Agricultural Research, Hebbal, Bangalore 560024, India

## Abstract

Brucellosis is one of the most important reemerging zoonoses in many countries. Brucellosis is caused by Gram-negative coccobacillus belonging to genus *Brucella*. Human brucellosis often makes the diagnosis difficult. The symptoms and clinical signs most commonly reported are fever, fatigue, malaise, chills, sweats headaches, myalgia, arthralgia, and weight loss. Some cases have been presented with only joint pain, lower backache, and involuntary limb movement, burning feet, or ischemic heart attacks. The focus of this work was to develop a highly sensitive and specific indirect ELISA by using smooth lipopolysaccharide antigen of *Brucella abortus 99* to detect anti-*Brucella* antibodies at Project Directorate on Animal Disease Monitoring and Surveillance. Serum samples collected from 652 individuals in whom fever was not the major symptom but the complaint was of joint pain, headache, lower backache, and so forth, were screened by Rose Bengal plate agglutination test (RBPT) and standard tube agglutination test (STAT). Subsequent testing of sera by indigenous indirect ELISA detected 20 samples positive (3.6% seroprevalence), and indirect ELISA was found to be more sensitive than RBPT and STAT. The seroprevalence in South Karnataka was 2.14%, and in North Karnataka it was 0.92%.

## 1. Introduction

Brucellosis is a disease of domestic and wild animals that can be transmitted to humans (zoonosis). The disease exists worldwide, particularly in the Mediterranean basin, the Arabian Peninsula, the Indian subcontinent, and in parts of Mexico, Central, and South America. Consumption of contaminated foods and occupational contact remain the main sources of infection [1]. Brucellosis is a recognised public health problem with worldwide distribution and one of the major causes of morbidity. It is also a disease of considerable economic and social importance. The *Brucella* species, particularly *Brucella melitensis* and *Brucella suis* are potential agents of biological terrorism [2, 3]. Brucellosis has been present for millennia [4]. A high prevalence in certain geographic areas is well recognized, although largely underestimated. More than 500,000 new cases are reported each year, and according to the World Health Organization, this figure underestimates the magnitude of the problem [5]. 

In this era of international tourism, brucellosis has become a common imported disease in the developed world [6]. Brucellosis is known to cause debilitating conditions if not promptly treated. Brucellosis is a clinically enigmatic disease. Insidious onset, undulating symptoms, and protean manifestations often make the diagnosis difficult. 

 The study done in Nigeria confirmed the endemicity of brucellosis, especially bovine brucellosis among slaughtered cattle at the abattoir, hence making it a source of occupational hazard to workers who are directly involved in cattle meat processing [7] Public health enlightenment should be focused on the zoonotic aspect of this disease as it relates to consumption of unpasteurised milk and other food items obtained from diseased animals. The occurrence of brucellosis in India was first established early in the previous century and since then has been reported from almost all states [8]. Vaishnavi and Kumar in 2007 [9] carried out a study on seroprevalence of brucellosis in Chandigarh among 292 blood donors and reported 6.36% of seroprevalence.

Milk and milk products are common sources of infection. The survival of *Brucella *may be prolonged in milk stored at optimal conditions to prevent souring. Milk may also neutralise the gastric acid and in turn protects ingested bacteria in the stomach. The raw milk, clotted cream, and unevenly heated milk can harbor live *Brucella *organisms [3].

*Brucellae *are capable of prolonged survival in the environment, so that viable organisms inhaled in dust may be infective. Blood transfusion, bone marrow transplantation, and possible kidney transplantation are sources of infection. Sexual transmission in semen may occur [8, 10].

In Germany, brucellosis has emerged as a disease among Turkish immigrants. In this population group, the infection is associated with major diagnostic delays, possible resulting in treatment failures, relapses, chronic courses, focal complication, and a high case-fatality rate [9]. A recent study done in Thassos of Greece showed that brucellosis is a disease of public health priority in Greece [11]. 

Brucellosis is a multisystem disease that may present with a broad spectrum of clinical manifestations. While hepatic involvement in brucellosis is not rare, it may rarely involve the kidney or display with cardiac manifestations. Central nervous system involvement in brucellosis sometimes can cause demyelinating syndromes [7]. Low back ache arthralgia or even arthritis of one or more big joints is common. The symptoms may be continuous or intermittent, and physical findings may be minimal. 

The prevalence of human brucellosis is difficult to estimate as many cases remain undiagnosed or misdiagnosed as pyrexia of unknown origin because either they are inapparent or of their protean manifestations. There is presently a wide battery of serological tests, which can be used for diagnosis of human brucellosis, although they each have important limitations. Keeping this view in mind, the present work is undertaken to study the seroepidemiology of human brucellosis in different parts of Karnataka State, India to know the endemicity of the disease and also to carry out the standardisation of an indirect Enzyme-linked immunosorbant assay (ELISA) for the serodiagnosis of human brucellosis to achieve an advancement in the disease diagnostics and also to make a comparative study of the standardized indirect ELISA with the conventional techniques like Rose Bengal agglutination test and standard tube agglutination test.

## 2. Materials and Methods

Serum samples were collected from 652 individuals in whom the fever was not the prominent symptom but had a history of joint pain, arthritis, backache, and shoulder pain, and so forth. A detailed history of these individuals was collected which included their name, age, occupation, nature of work, history of consumption of raw milk, history of fever (nature and duration) in the past, and complaints of joint pain, if any. These samples consisted of 333 RA-negative sera, 176 ASLO negative sera, and 143 CRP-negative sera. The collected sera were negative for the Rheumatoid Arthritis, C-reactive protein, and Anti-streptolysin O tests. 

The serum samples were analyzed in three phases. In the first phase RBPT was performed. In the second phase, the seropositive samples were analyzed by STAT. The antigens required for both tests were procured from Institute of Animal Health and Veterinary Biologicals, Bangalore. In the third phase, indirect ELISA by using smooth lipopolysaccharide of *Brucella abortus 99* was employed. Data were recorded and analysed for the interpretation of the results to know the influence of age and sex in contracting brucellosis. The data was further used to know the endemicity of the disease in different parts of Karnataka State.

**Procedure for Indirect ELISA**

  Antigen: SLPS (smooth lipopolysaccharide) of *Brucella abortus *99 purified by hot phenol water extraction method [12] was used.

The serum collected from confirmed cases of brucellosis (isolated on repeating sampling and blood culturing) obtained from a Medical College, Bijapur (Courtesy Dr. Mantur) was used as strong positive serum control. 

The serum sample taken from the healthy individuals was used as the negative control serum.

The moderate positive control was prepared by diluting the strong positive serum in negative serum (1 : 20 diluted).

Reagents were procured commercially to develop the indirect ELISA. The rabbit antihuman HRP conjugate (Bangalore Genie), O-Phenylene Diamine Dihydrochloride (OPD), hydrogen peroxide (H_2_O_2_), bovine gelatin (British Pharmacopoeia grade 4), and 96 well ELISA plate (Nunc polysorp) were used. Optimal working dilutions of SLPS antigen, control sera, and rabbit antihuman HRP conjugate were established by a checkerboard titration for use in indirect ELISA. Control and test sera were used at 1 : 100 dilutions. 

Microtiter plates were coated with SLPS antigen of *Brucella abortus 99* at 100 *μ*L per well (10 ng) in carbonate bicarbonate buffer (pH 9.6 + 0.05) separately and were incubated overnight at 4°C. The plates were then washed three times with washing buffer consisting of 0.002 M phosphate-buffered saline (PBS). Test and control sera were diluted (1 : 100) in blocking buffer (1% bovine gelatin in 0.01 M PBS and 0.1% Tween 20) and then added to respective wells of the plate in duplicate for test sera and quadruplicate for control sera (100 *μ*L volume). Samples were incubated for 1 hr at 37°C, and the plates were then washed as described above. The rabbit antihuman IgG HRP conjugate (1 : 3000) diluted in blocking buffer was then added to all the wells (100 *μ*L) and incubated for 1 hr at 37°C. The plates were then washed and treated with 100 *μ*L of freshly prepared OPD solution with H_2_O_2_ for 10 minutes. Finally 100 *μ*L of 1 M sulphuric acid per well was added to stop the enzyme substrate reaction. The plates were read at 492 nm, using an ELISA Microtiter plate reader.

There are a number of methods for determination of seropositive or seronegative thresholds. Based on the results obtained after screening large number of samples at the PD_ADMAS laboratory using the indirect ELISA, we have recommended that a positive result can be considered when the ELISA positive-negative ratio is ≥3 [13] and three standard deviation of positive and negative OD values. This means that any sample that gives an OD value three times more than the OD value of the negative control is positive and that below is negative.

## 3. Result

When subjected for the serological analysis, out of 652 samples none were positive by RBPT and STAT, but 20 samples had come positive by indirect ELISA.

The overall seroprevalence of brucellosis in this category of individuals is 3.06% by indirect ELISA. The statistical analyses of the samples have shown that the seroprevalence is high (1.99%) in the age group of 30–40 years (13 seropositive out of 652) followed by (1.07%) in the age group >40 years (7 seropositive out of 652) and the same is depicted in Table 1. 

Table 2 depicts the geographical distribution of seropositive and negative cases in North and South Karnataka. The respective percentage in South Karnataka is 2.14% (14 seropositive out of 652) and 0.92% (6 sero positive out of 652) in North Karnataka.

The endemicity of brucellosis in different parts of South Karnataka is depicted in Table 3, and the endemicity of brucellosis in different parts of North Karnataka is depicted in Table 4. 

## 4. Discussion

Among the individuals with no history of fever, the seropositivity is found to be 3.06%.The sero positivity is detected only by indirect ELISA indicating the need of tests with higher sensitivity as no cases are detected positive by RBPT and STAT in this group. The geographical distribution of brucellosis in South Karnataka is found to be 2.14% and North Karnataka 0.92%. The sero prevalence is 2.3% in male and 0.76% in female; ratio being 2.33 : 1. The statistical analyses of the samples have shown that the sero prevalence is high in the age group 30–40 (1.99%) followed by age group >40 years (1.07%). 

The diagnosis of brucellosis remains as one of the most challenging tests of medical knowledge and clinical acumen of the physicians.

ELISA is a rapid, sensitive, and specific assay providing a profile of immunoglobulin classes in the diagnosis of acute and chronic brucellosis; therefore, it is useful for mass screening and could be considered the method of choice for the serological diagnosis of the named disease [14].

In some cases, persistence of the infection cannot be confirmed with positive cultures and clinical findings, and serological tests may play an important role in such circumstances. Serological tests measuring specific antibodies to *Brucella *lipopolysaccharide are of great importance in the initial diagnosis of the disease [15].

It is difficult to compare seroprevalence of brucellosis in different studies as it varies from place to place and time to time. Magnitude of problem differs from state to state in India. Even with in the states in which prevalence is known it differs from place to place. The diagnosis of brucellosis also depends upon type of antigen, diagnostic techniques used, and on levels of antibody titers considered as diagnostic. Selection criteria used for selection of cases for laboratory investigation for brucellosis also play an important role in determining seroprevalence of brucellosis in particular geographical area. Chadda et al. [16] reported an incidence of brucellosis in individuals who had habit of raw meat ingestion.

The most common localized complication of brucellosis is joint infection which may affect large- or medium-sized peripheral joints, sacroiliac joints, or the spine. The clinical manifestations may vary from subclinical disease which may pass unnoticed to a disease with general symptoms such as fever, diaphoresis, lymphadenopathy, anorexia, and malaise. Very severe localized disease includes endocarditis or central nervous system effects. The possibility of contracting brucellosis during travel through endemic areas or through illegally imported products has to be kept in mind [17–19]. The clinical manifestation of brucellar arthritis is nonspecific, and only searching for it when suspicion of exposure exists may lead to its search and diagnosis. 

Although the definite diagnosis of brucellosis requires the isolation of the organism from blood or other body fluids, since brucellae are slow growing organisms and require special culture conditions [20] owing to the delay in the isolation, serological methods are required for a rapid diagnosis. The antibodies detected by serological testing are directed against the lipopolysaccharide of the bacterial cell wall [21].

Virtually any organ or system can be involved with brucellosis, and localization of the process may cause focal symptoms. The most common sites for localization are osteoarticular; large weight bearing joints are involved more often than small joints.

In our study, the sero positivity was detected only by indirect ELISA by using SLPS of *Brucella abortus 99.* The ELISA test is reported to be rapid, highly sensitive, and specific for detecting the *Brucella*-specific IgG, IgM, and IgA antibodies in blood and cerebrospinal fluid [22]. 

Brucellosis has a worldwide distribution with different rate of focal disease involving any organ. Clinical manifestations and severity of symptoms may vary depending on geographic areas with respect to pathogenic species of *Brucella* and the host. 

Nagalotimath et al., 1985 [23] have reported 30% diagnosed cases, which are not linked to any high-risk occupation in 1978. In India, Mathur in 1954 [1] has reported many outbreaks of brucellosis in families and Institute, which he attributed to consumption of raw milk and ice cream.

If a seroepidemiological survey was made by using RBPT and STAT only, all cases would have been declared negative. ELISA test has the advantage of being highly sensitive and specific. Indirect ELISA used in the present study detected positive cases of brucellosis. An occupational exposure can result through consumption of raw milk or milk products and even handling of meat. In the present study, the results obtained suggested that the alertness of clinicians and close collaboration with microbiologists are essential even in endemic areas to diagnose and treat brucellosis.

These individuals have not provided any history of consumption of unpasteurized milk and milk products and had no risk of developing brucellosis, but the chances of animal contact could not be ruled out as some of the individuals were from the rural areas. In view of the limited efficacy of the vaccine, maintaining the hygiene especially while being associated with the livestock and consumption of properly processed milk and milk products is the only way to prevent brucellosis among the general human population.

## Figures and Tables

**Table 1:** Influence of age on seropositive results of brucellosis.

Age	Seropositive male	Seropositive female	Total	Seronegative male	Seronegative female	Total
30–40	11	02	13	218	220	451
>40	07	00	07	101	93	201

Total	18	02	20	319	313	652

**Table 2:** Geographical distribution of brucellosis in South Karnataka and North Karnataka.

Place	Seropositive males	Seropositive females	Total	Seronegative males	Seronegative females	Total
South Karnataka	10	04	14	337	192	543
North Karnataka	04	02	06	63	40	109

Total	14	06	20	400	232	652

**Table 3:** Endemicity of brucellosis in South Karnataka.

Place	Male	Percentage (Taken for 20)	Female	Percentage (Taken for 20)	Total	Percentage (Taken for 20)
Bangalore	00	00	00	00	00	00
Mandya	10	50	00	00	10	50
Mysore	00	00	2	10	2	10
Tumkur	00	00	2	10	2	10
Mudirangadi	00	00	00	00	00	00

Total	10	50	04	20	14	70

**Table 4:** Endemicity brucellosis in North Karnataka.

Place	Male	Percentage (Taken for 20)	Female	Percentage (Taken for 20)	Total	Percentage (Taken for 20)
Hospet	02	10	00	00	02	10
Bellary	02	10	00	00	02	10
Koppal	00	00	01	05	01	05
Bijapur	00	00	01	05	01	05

Total	04	20	02	10	06	30
